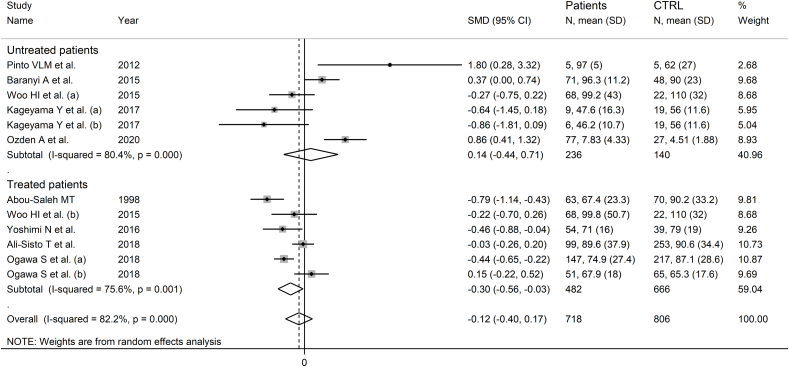# Arginine metabolomics in mood disorders

**DOI:** 10.1016/j.heliyon.2024.e27292

**Published:** 2024-03-14

**Authors:** Angelo Zinellu, Sara Tommasi, Stefania Sedda, Arduino A. Mangoni

**Affiliations:** a Department of Biomedical Sciences, University of Sassari, Sassari, Italy; b Department of Clinical Pharmacology, Southern Adelaide Local Health Network, Australia; c Discipline of Clinical Pharmacology, Flinders University, Adelaide, Australia

**Keywords:** Arginine, NO, ADMA, SDMA, Citrulline, Ornithine, Mood disorder, Bipolar disorder

## Abstract

Alterations of nitric oxide (NO) homeostasis have been described in mood disorders. However, the analytical challenges associated with the direct measurement of NO have prompted the search for alternative biomarkers of NO synthesis. We investigated the published evidence of the association between these alternative biomarkers and mood disorders (depressive disorder or bipolar disorder). Electronic databases were searched from inception to the June 30, 2023. In 20 studies, there was a trend towards significantly higher asymmetric dimethylarginine (ADMA) in mood disorders vs. controls (p = 0.072), and non-significant differences in arginine (p = 0.29), citrulline (p = 0.35), symmetric dimethylarginine (SDMA; p = 0.23), and ornithine (p = 0.42). In subgroup analyses, the SMD for ADMA was significant in bipolar disorder (p < 0.001) and European studies (p = 0.02), the SMDs for SDMA (p = 0.001) and citrulline (p = 0.038) in European studies, and the SMD for ornithine in bipolar disorder (p = 0.007), Asian (p = 0.001) and American studies (p = 0.005), and patients treated with antidepressants (p = 0.029). The abnormal concentrations of ADMA, SDMA, citrulline, and ornithine in subgroups of mood disorders, particularly bipolar disorder, warrant further research to unravel their pathophysiological role and identify novel treatments in this group (The protocol was registered in PROSPERO: CRD42023445962)

## 1 Introduction

Despite significant advances, largely based on the monoamine hypothesis which was formulated more than 50 years ago [1], the treatment failure in patients suffering from the two main subtypes of mood disorder, depressive disorder and bipolar disorder, remains unacceptably high, with negative implications for the community and healthcare systems [[2], [3], [4], [5]]. This vexing issue is likely the result of the incomplete knowledge regarding the pathophysiological mechanisms involved in mood disorders, which prevents the discovery of new disease biomarkers, druggable targets, and more effective therapies [[6], [7], [8], [9]].

In the ongoing search for breakthrough molecular mechanisms, studies have sought to determine the pathophysiological role of nitric oxide (NO), synthetised by three NO synthase (NOS) isoforms (Fig. 1) [10,11]. All NOS isoforms modulate cell signalling in the brain, particularly the neuronal isoform, nNOS, which co-locates with key receptors [12,13], and is also influenced by muscarinic and purinergic receptors and by serotoninergic pathways [[14], [15], [16]]. NO, itself can nitrosylate several proteins, influencing their activity and regulating neuronal function and local blood flow [[17], [18], [19]]. Despite the various homeostatic effects exerted by NO experimental and human studies have provided conflicting results regarding whether mood disorders are associated with an increased or a decreased synthesis and availability of NO [10,11,20,21]. This issue is particularly relevant given that alterations in NO concentrations can either exert beneficial effects on cellular homeostatic mechanisms or toxicity [22,23]. However, the high reactivity of NO has traditionally represented a major barrier to its measurement in biological samples [24,25]. The measurement of alternative metabolites, e.g., nitrite and nitrate, is also affected by a number of factors that have curtailed its routine use in research and clinical studies [26,27].

**Fig. 1:**
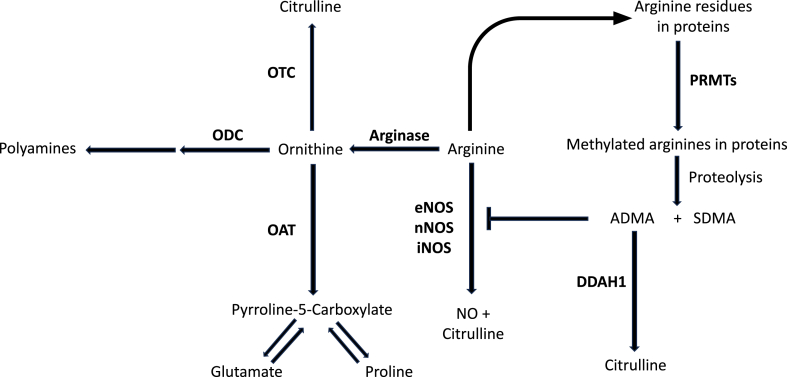
Arginine metabolic pathways. PRMTs, protein arginine methyltransferases; ADMA, asymmetric dimethylarginine; SDMA, symmetric dimethylarginine; DDAH1, isoform 1 of dimethylarginine dimethylaminohydrolase; eNOS, endothelial nitric oxide synthase; nNOS, neuronal nitric oxide synthase; iNOS, inducible nitric oxide synthase; NO, nitric oxide; OAT, ornithine aminotransferase; ODC, ornithine decarboxylase; OTC, ornithine transcarbamylase.

To address this issue, a number of metabolites within the arginine biochemical pathways have been investigated in health and disease states given their direct or indirect effects in modulating NO synthesis (Fig. 1) [[28], [29], [30], [31], [32], [33]]. Their assessment has also been proposed to further investigate the role of NO and arginine pathways in mood disorders [29,34].

Therefore, we critically appraised the available evidence of the association between these arginine metabolites, mood disorders, and specific clinical and demographic characteristics, including the type of mood disorder (depressive vs. bipolar) [35], and the use of antidepressant treatment. Our initial hypothesis was that mood disorders were associated with abnormalities in one or more arginine metabolites when compared to healthy controls.

## 2 Methods

### 2.1 Literature search

Two independent investigators searched the electronic databases until the June 30, 2023 using the search strategy described in Supplementary Tab. 1 and the following inclusion criteria: the measurement of arginine, asymmetric (ADMA) and symmetric (SDMA) dimethylarginine, citrulline, or ornithine in adult patients with mood disorder and healthy subjects in fully available articles written in English. Individual articles’ references were hand searched for further articles.

First author, publication year, study country, sample size, age, male to female ratio, use of antidepressant medications, and analytical method used for arginine metabolomics evaluation were extracted from each article.

The risk of bias and the certainty of evidence were assessed using standard methods [36,37], the PRISMA 2020 statement was adhered to (Supplementary Tab. 1) [38], and the review was registered (PROSPERO registration number: CRD42023445962).

### 2.2 Statistical analysis

Forest plots were generated to investigate differences between patients suffering from mood disorder and controls using standard mean differences (SMDs) [39,40]. Heterogeneity was assessed using the Q statistic [41,42]. Sensitivity analysis assessed whether the pooled SMD values were stable [43], and standard methods were used to assess publication bias [[44], [45], [46]]. We further investigated associations between the effect size and pre-defined patient and study characteristics. Statistical analyses were conducted using Stata 14 (Stata Corp., College Station, TX, USA).

## 3 Results

### 3.1 Screening process

The study selection is described in Fig. 2. Of the 4168 articles initially identified, 4145 were excluded. Of the remaining 23 articles, a further three were excluded, leaving 20 studies [[47], [48], [49], [50], [51], [52], [53], [54], [55], [56], [57], [58], [59], [60], [61], [62], [63], [64], [65], [66]] (Table 1). Nineteen studies had a low risk of bias [[47], [48], [49],[51], [52], [53], [54], [55], [56], [57], [58], [59], [60], [61], [62], [63], [64], [65], [66]], and the remaining one had a moderate risk of bias [50] (Supplementary Tab. 2). The cross-sectional study design reduced the initial certainty of evidence to low.

**Fig. 2:**
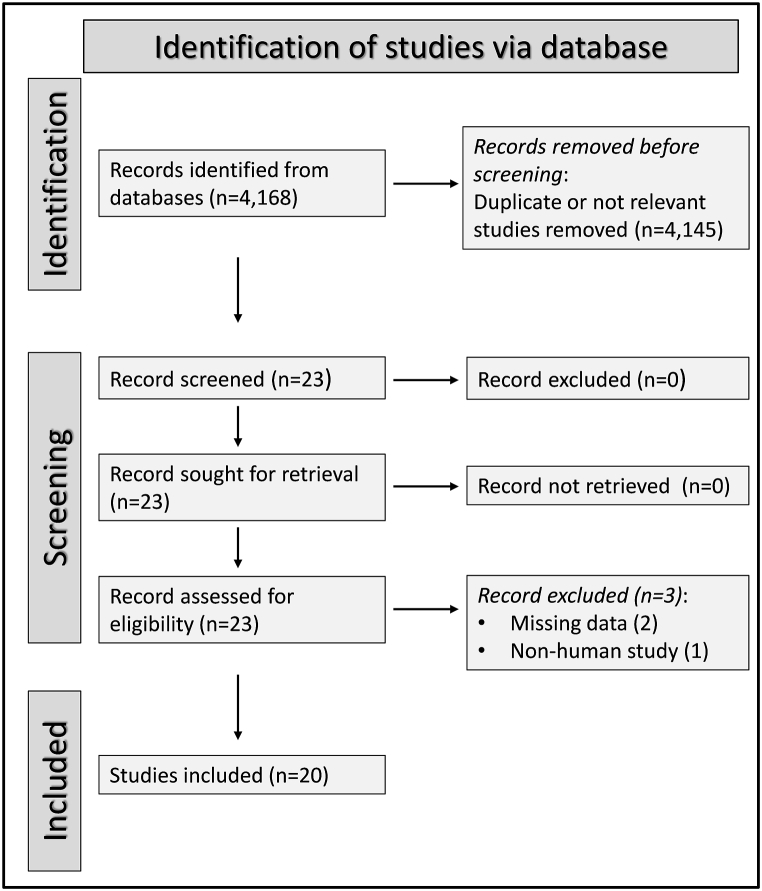
PRISMA 2020 flow diagram.

**Table 1:** Study characteristics.

**Study**	Healthy controls					Patients with depressive disorder				
	**n**	**Age** (Years)	**M/F**	**Arginine** **ADMA** **SDMA** (Mean ± SD)	**Citrulline** **Ornithine** (Mean ± SD)	**n**	**Age** (Years)	**M/F**	**Arginine** **ADMA** **SDMA** (Mean ± SD)	**Citrulline** **Ornithine** (Mean ± SD)
Abou-Saleh MT et al., 1998, UAE [47]	38	36	0/38	65.4 ± 24.13 NR NR	NR 90.2 ± 33.2	30	35	0/30	89.1 ± 30.5 NR NR	NR 67.4 ± 23.3
Maes M et al., 1998, Belgium [48]	15	48	5/10	137 ± 28 NR NR	NR NR	35	51	18/17	124 ± 22 NR NR	NR NR
Mauri MC et al., 1998, Italy [49]	28	42	16/12	87.6 ± 30.9 NR NR	NR NR	29	47	15/14	83.5 ± 100.6 NR NR	NR NR
Mitani H et al., 2006, Japan [50]	31	41	17/14	93.2 ± 42.8 NR NR	NR NR	23	38	11/12	116.8 ± 54.8 NR NR	NR NR
Pinto VL et al., 2012, Brazil [51]	5	NR	NR	130 ± 19 NR NR	NR 62 ± 27	5	NR	NR	104 ± 9 NR NR	NR 97 ± 5
Canpolat S et al., 2014, Turkey [52]	44	27	14/30	NR 6.8 ± 4.0 NR	NR NR	41	26	13/28	NR 7.3 ± 5.3 NR	NR NR
Baranyi A et al., 2015, UK [53]	48	40	31/17	96.1 ± 13.8 0.61 ± 0.08 0.71 ± 0.15	30.6 ± 7.6 90 ± 23	71	50	48/23	100.2 ± 19.8 0.64 ± 0.09 0.64 ± 0.11	29.9 ± 6.2 96.3 ± 11.2
Woo HI et al. (a), 2015, Korea [54]	22	68	4/18	57.7 ± 26.7 NR NR	30.9 ± 8.1 110 ± 32	68	65	16/22	70.9 ± 33.4 NR NR	34.9 ± 15.1 99.2 ± 43
Woo HI et al. (b), 2015, Korea [54]	22	68	4/18	57.7 ± 26.7 NR NR	30.9 ± 8.1 110 ± 32	68	65	16/22	77.1 ± 33.1 NR NR	36.1 ± 19.3 99.8 ± 50.7
Yoshimi N et al., 2016, Japan [55]	39	36	39/0	122 ± 19 NR NR	37.0 ± 6.4 79 ± 19	54	41	54/0	131 ± 23 NR NR	38.0 ± 7.1 71 ± 16
Hess S et al., 2017, Canada [56]	36	26	20/16	84.9 ± 25.2 NR NR	35.2 ± 6.8 NR	35	27	20/15	73.5 ± 21.5 NR NR	31.6 ± 6.0 NR
Kageyama Y et al. (a), 2017, Japan [57]	19	36	10/9	102 ± 22 NR NR	31.9 ± 10.9 56 ± 11.6	9	39	3/6	85 ± 18 NR NR	28.9 ± 12.4 47.6 ± 16.3
Kageyama Y et al. (b), 2017, Japan [57]	19	36	10/9	102 ± 22 NR NR	31.9 ± 10.9 56 ± 11.6	6	42	1/5	79 ± 19 NR NR	21.4 ± 5.1 46.2 ± 10.7
Ali-Sisto T et al., 2018, Finland [58]	253	55	124/129	118 ± 35 0.79 ± 0.34 1.43 ± 0.61	28.2 ± 8.7 90.6 ± 34.4	99	39	43/56	99 ± 24 0.77 ± 0.29 1.27 ± 0.50	27.4 ± 10 89.6 ± 37.9
Moaddel R et al., 2018, USA [59]	25	NR	NR	NR NR NR	23.8 ± 7.22 NR	29	NR	NR	NR NR NR	19.25 ± 5.17 NR
Ogawa S et al. (a), 2018, Japan [60]	217	41	100/117	79.2 ± 25.7 NR NR	35.3 ± 8.6 87.1 ± 28.6	147	42	74/73	77.9 ± 21.9 NR NR	36.1 ± 8.6 74.7 ± 27.4
Ogawa S et al. (b), 2018, Japan [60]	65	43	20/45	90.6 ± 28.3 NR NR	32.2 ± 10.3 65.3 ± 17.6	51	44	27/24	83.7 ± 24.1 NR NR	31.9 ± 11.6 67.9 ± 18.0
Yilmaz E et al., 2019, Turkey [61]	30	NR	13/17	0.34 ± 0.23* NR NR	NR NR	30	NR	13/17	0.63 ± 0.41* NR NR	NR NR
Ozden A et al., 2020, USA [62]	27	38	7/20	1.4 ± 1.3 0.16 ± 0.08 0.01 ± 0.01	4.4 ± 1.0 4.5 ± 1.9	77	41	28/49	6.6 ± 7.2 0.1 ± 0.03 0.02 ± 0.01	5.2 ± 1.8 7.83 ± 4.33
Ustundag MF et al., 2020, Turkey [63]	30	35	12/18	74.7 ± 9.2 NR 3.67 ± 0.98	NR NR	30	35	13/17	91.6 ± 18.8 NR 4.52 ± 0.85	NR NR
Bilbao AV et al., 2021, USA [64]	30	NR	NR	NR 0.42 ± 0.07° 0.36 ± 0.05°	NR NR	30	NR	NR	NR 0.42 ± 0.09° 0.41 ± 0.05°	NR NR
Braun D et al. (a), 2021, Germany [65]	16	31	8/8	NR 82 ± 12.6* NR	NR NR	14	52	9/5	NR 151 ± 36.9* NR	NR NR
Braun D et al. (b), 2021, Germany [65]	16	31	8/8	NR 82 ± 12.6*	NR NR	44	50	27/17	NR 138 ± 39* NR	NR NR
Loeb E et al., 2022, France [66]	895	40	457/438	81.8 ± 19.5 NR NR	30.1 ± 8.1 NR	460	46	145/315	90.9 ± 22.8 NR NR	27.2 ± 8.0 NR

Legend: ADMA, asymmetric dimethylarginine; F, female; M, male; NR, not reported; SDMA, symmetric dimethylarginine.

The concentration is expressed in μmol/L except where otherwise indicated. *, ng/mL; °, pg/mL.

### 3.2 Arginine

Sixteen studies including 19 group comparators reported arginine in 1327 patients with mood disorders and 1839 controls (Table 1) [[47], [48], [49], [50], [51],[53], [54], [55], [56], [57], [58],[60], [61], [62], [63],^66^]. Fifteen study groups included patients with depressive disorder and four with bipolar disorder. Eight study groups included patients receiving antidepressants, ten untreated patients, whereas relevant information was not available in one. Fifteen studies had a low risk of bias and one moderate (Supplementary Tab. 2).

The forest plot showed that the serum concentrations of arginine in mood disorders were similar to controls (Fig. 3). Sensitivity analysis confirmed the stability of the results (Supplementary F. 1). No significant publication bias was observed (Supplementary F. 2).

**Fig. 3:**
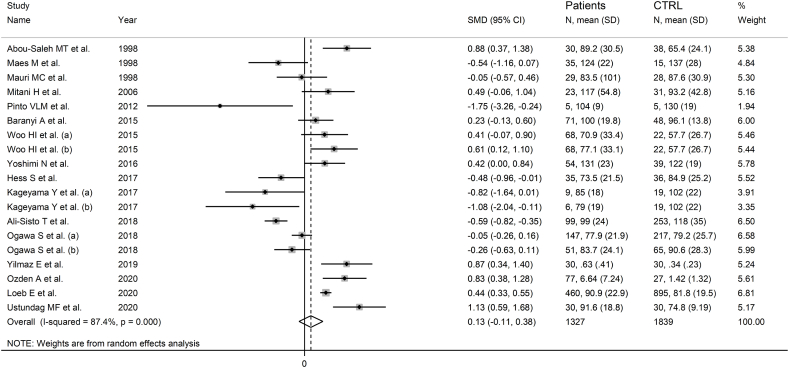
Forest plot of arginine concentrations in patients with mood disorders and healthy controls.

No significant associations were observed between the SMD and age (t = 0.17, p = 0.87), sex distribution (t = −0.91, p = 0.38), study size (t = 0.37, p = 0.72), or publication year (t = 0.22, p = 0.83). The pooled SMD were similar (p = 0.30) in studies in depressive disorder and bipolar disorder (Supplementary F. 3), between Asian, European, and American studies (p = 0.25; Supplementary F. 4), and between studies using liquid chromatography and other methods (p = 0.67) (Supplementary F. 5). Among liquid chromatography studies, the pooled SMD was similar (p = 0.58) in studies using fluorimetric detection and mass spectrometry (Supplementary F. 6). Finally, the effect size was similar (p = 0.22) in studies in treated and untreated patients (Supplementary F. 7).

The high and unexplainable heterogeneity led to the downgrading of the level of certainty to very low.

### 3.3 Asymmetric dimethylarginine

Seven studies including eight group comparators reported ADMA in 406 patients with mood disorders and 464 controls (Table 1) [52,53,58,[62], [63], [64], [65]]. Five studies included participants with depressive disorder and two with bipolar disorder. Three study groups included patients receiving antidepressants, three untreated patients, whereas no information was available in two. All studies had a low risk of bias (Supplementary Tab. 2).

The forest plot showed that patients with mood disorders had a trend towards statistically higher ADMA concentrations compared to controls (Fig. 4). The direction of the pooled SMD remained consistent in sensitivity analysis (Supplementary F. 8).

**Fig. 4:**
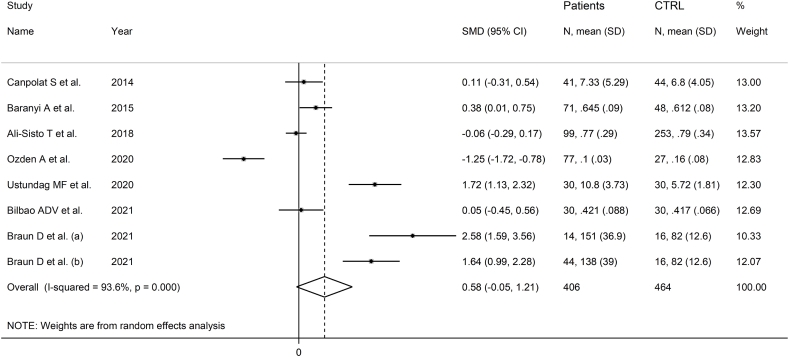
Forest plot of ADMA concentrations in patients with mood disorders and healthy controls.

Publication bias and meta-regression could not be evaluated because of the insufficient number of studies for these types of analyses. There was a significant difference (p = 0.037) in the pooled SMD between studies in depressive disorder and bipolar disorder (Fig. 5) with the bipolar subgroup exhibiting a lower heterogeneity. In addition, the pooled SMD was statistically significant in European, but not in Asian or American studies (Supplementary F. 9), whereas there were no significant differences between studies of treated and untreated patients (Supplementary F. 10).

**Fig. 5:**
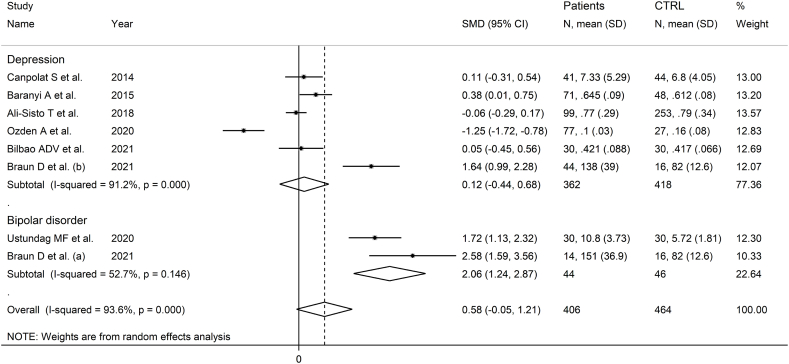
Forest plot of ADMA concentrations in patients with mood disorders and healthy controls according to disease type (depressive disorder vs. mood disorder).

The lack of evaluation of publication bias led to the downgrading of the level of certainty to very low.

### 3.4 Symmetric dimethylarginine

Five studies investigated SDMA in 307 patients with mood disorders and 388 controls (Table 1) [53,58,[62], [63], [64]]. Patients with depressive disorder were investigated in four studies and with bipolar disorder in the remaining one. In one study group patients were receiving antidepressants, in two patients were untreated, whereas in the remaining two no information regarding treatment was provided. All studies had low risk of bias (Supplementary Tab. 2).

The forest plot showed that SDMA was similar between patients with mood disorders and controls (Fig. 6). The corresponding pooled SMD was stable (Supplementary F. 11).

**Fig. 6:**
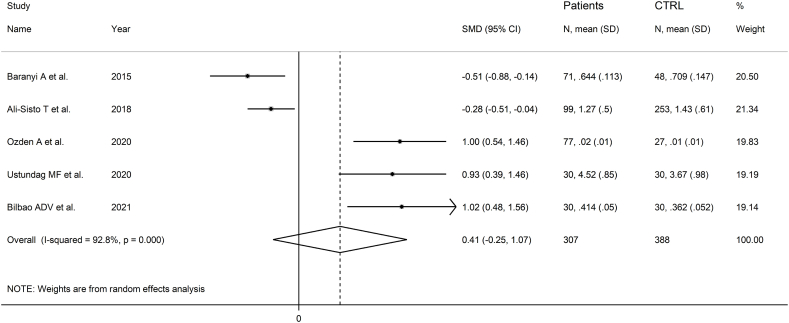
Forest plot of SDMA concentrations in patients with mood disorders and healthy controls.

We could not assess publication bias or perform meta-regression analysis because of the insufficient number of selected studies. There was a significant difference (p = 0.02) in effect size between European and Asian studies (Supplementary F. 12), with a substantial reduction in heterogeneity in both subgroups.

Similar to ADMA, the lack of evaluation of publication bias led to the downgrading of the level of certainty to very low.

### 3.5 Citrulline

Ten studies including 13 group comparators reported citrulline in 1174 patients with mood disorders and 1687 healthy controls (Table 1) [[53], [54], [55], [56], [57], [58], [59], [60],^62^,^66^]. Eleven study groups included patients with depressive disorder and the remaining two bipolar disorder. Eight study groups included untreated patients and the remaining five patients receiving antidepressants. All studies had low risk of bias (Supplementary Tab. 2).

The forest plot showed that citrulline was similar between the two groups (Fig. 7), with stable pooled SMD values (Supplementary F. 13). There was no publication bias. The addition of one missing study with the “trim-and-fill” method did not substantially change the effect size (Supplementary F. 14).

**Fig. 7:**
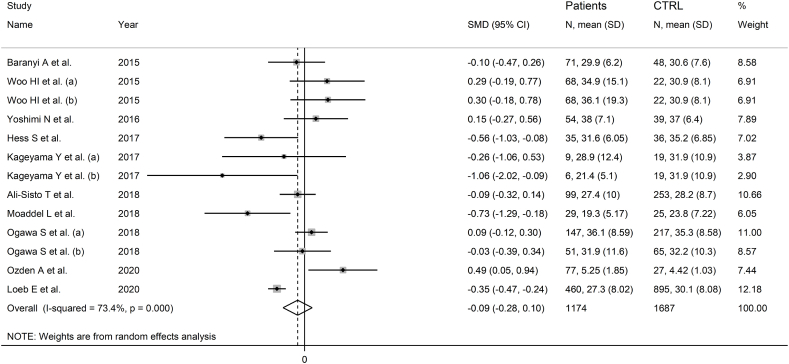
Forest plot of citrulline concentrations in patients with mood disorders and healthy controls.

No associations were observed between the effect size and age of participants (t = −0.63, p = 0.59), year of publication (t = −0.54, p = 0.60), and sample size (t = −0.68, p = 0.51). However, a significant association was observed with sex distribution (Supplementary F. 15A and 15B).

The pooled SMD was similar (p = 0.74) in studies in depressive disorder and bipolar disorder (Supplementary F. 16). The pooled SMD was significant in European, but not in Asian or American studies (Supplementary F. 17), with a lower heterogeneity in the Asian subgroup. The pooled SMD was similar (p = 0.64) in studies using liquid chromatography and capillary electrophoresis (Supplementary F. 18). In studies using liquid chromatography, the pooled SMD was similar (p = 0.31) in studies with fluorimetric detection and mass spectrometry detection (Supplementary F. 19). The pooled SMD was also similar (p = 0.19) between studies in patients receiving antidepressants and untreated patients (Supplementary F. 20).

The final level of certainty was unchanged (low).

### 3.6 Ornithine

Nine studies including 12 group comparators investigated ornithine in 718 patients with mood disorders and 806 healthy controls (Table 1) [47,51,[53], [54], [55],^57^,^58^,^60^,^62^]. Ten study groups included patients with depressive disorder and the remaining two with bipolar disorder. Six study groups included untreated patients and the remaining six patients receiving antidepressants. All studies had low risk of bias (Supplementary Tab. 2).

The forest plot showed that ornithine was similar in patients with mood disorders and controls (Fig. 8). No significant deviations in pooled SMD were observed in sensitivity analysis (Supplementary F. 21) and no publication bias was detected (Supplementary F. 22).

**Fig. 8:**
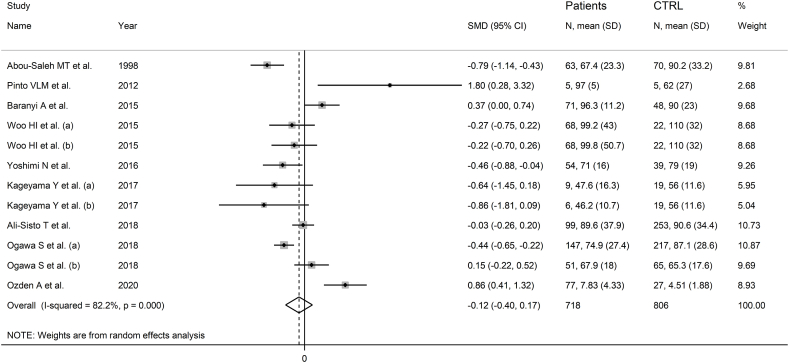
Forest plot of studies examining ornithine concentrations in patients with mood disorders and healthy controls.

No significant associations were observed between the SMD and participant age (t = 0.19, p = 0.85), sex distribution (t = 0.60, p = 0.56), publication year (t = 1.26, p = 0.24), or participant number (t = −0.32, p = 0.76). The pooled SMD was significant in studies of bipolar disorder but not depressive disorder (Fig. 9), with a reduced heterogeneity in the bipolar subgroup. Moreover, the pooled SMD was significantly different in Asian and American, but not European studies (Supplementary F. 23), with a lower heterogeneity in the American subgroup. The SMD was also statistically significant using capillary electrophoresis but not liquid chromatography (Supplementary F. 24), with a lower heterogeneity in the capillary electrophoresis subgroup. In liquid chromatography studies, there was no significant difference (p = 0.43) in pooled SMD between fluorimetric detection and mass spectrometry detection (Supplementary F. 25), with absent between-study variance in the mass spectrometry subgroup. Finally, the pooled SMD was statistically significant in treated, but not untreated patients (Supplementary F. 26).

**Fig. 9:**
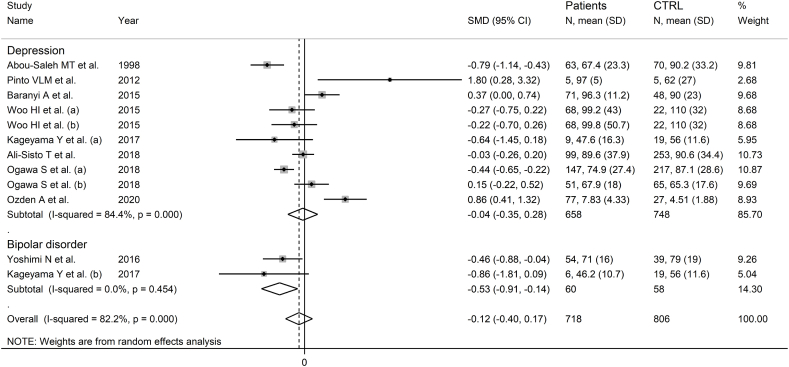
Forest plot of ornithine concentrations in patients with mood disorders and healthy controls according to disease type (depressive disorder vs. bipolar disorder).

The final level of certainty remained unchanged (low).

## 4 Discussion

This study has reported non-significant differences in key metabolites of the arginine pathways, arginine, ornithine, citrulline, and SDMA, between individuals suffering from mood disorders and healthy controls, and a trend towards higher ADMA concentrations in patients with mood disorders. However, in subgroup analysis ADMA was significantly higher in subjects with bipolar disorder vs. controls and in European studies. Opposite, significant effects were observed for SDMA in European vs. Asian studies, and significantly lower concentrations were observed with citrulline only in European studies. Furthermore, ornithine concentrations were significantly lower specifically in bipolar disorder and in subjects with mood disorders receiving antidepressants, whereas opposite, significant effects were observed in Asian vs. American studies. Additional subgroup differences for ornithine were observed according to the type of analytical method used. The overall absence of significant associations between the effect size of other metabolites and analytical method suggests the potential validity of several methodological approaches for the routine assessment of arginine metabolomics in research and clinical studies [26,67]. No further associations were observed barring a significant correlation between the SMD of citrulline and sex distribution.

ADMA and SDMA are formed following methylation of arginine contained in proteins (Fig. 1) [68,69]. After protein degradation, ADMA is either transformed to citrulline and dimethylamine by dimethylarginine dimethylaminohydrolase 1 (DDAH1) or transported into the extracellular compartment for remote DDAH1 metabolism or renal elimination [30,[70], [71], [72], [73]]. Differently from ADMA, SDMA is not metabolised by DDAH1 and undergoes renal elimination [28,71,74]. Both ADMA and SDMA have been shown to downregulate NO synthesis. However, whilst ADMA is a potent reversible inhibitor of all NOS isoforms (Fig. 1) [75,76], the interaction between SDMA and NOS is more controversial, with studies reporting that this methylated arginine can favour NOS uncoupling and inhibit arginine transport, reducing the amount of arginine as a NOS substrate [71,77,78]. Our subgroup analyses suggest a selective increase in circulating ADMA concentrations, with consequent reduction in NO synthesis, in bipolar depression. Although the relatively small number of studies captured in search warrants caution with data interpretation, this observation suggests that NO may have a different pathophysiological role in specific subtypes of mood disorder. Furthermore, the reported associations between the SMD of ADMA and geographical location and treatment with antidepressants suggest new avenues for research investigating the interplay between arginine metabolomics, NO, and mood disorders. Previous studies investigating the association between ADMA concentrations and specific ethnic groups have provided conflicting results, with some reports suggesting a link [[79], [80], [81], [82], [83]], while others reporting negative findings [84,85]. Regarding SDMA, the reported link between its circulating concentrations and ethnicity reported in other studies should also prompt further research to investigate the pathophysiological role of this indirect modulator of NO synthesis in mood disorders [83,84].

The role of citrulline in arginine metabolism is complex as this metabolite is a product of reactions catalysed by NOS, DDAH1, and ornithine transcarbamylase, as well as an argininosuccinate synthetase substrate in reactions leading to the synthesis of arginine (Fig. 1) [31]. Whilst no overall significant associations were observed between citrulline concentrations and mood disorders, the lower concentrations observed in European studies suggests the need for additional research, also given the results of recent studies in non-psychiatric populations reporting significant ethnic-related differences in citrulline concentrations [79,86]. Ornithine also plays a complex role within arginine metabolism, serving as both a substrate for ornithine aminotransferase for the synthesis of l-glutamate 5-semialdehyde and either glutamate or proline, for ornithine decarboxylase as the first step in the synthesis of polyamines, and for ornithine transcarbamylase which is involved in the synthesis of citrulline, and a product of arginase which uses arginine as substrate [31]. The significantly lower ornithine concentrations in bipolar, but not depressive disorder, suggest, similarly to ADMA, the presence of disease subtype-specific differences in the pathophysiological role of arginine metabolism. Such alterations could be secondary to reduced arginase activity and/or increased ornithine aminotransferase, ornithine decarboxylase, or ornithine transcarbamylase activity. A reduced arginase activity has been previously reported in bipolar disorder. In one study, the plasma arginase activity in 43 patients with bipolar disorder (male to female ratio: 33/10) was significantly lower compared to that measured in 31 age- and sex-matched control subjects (male to female ratio: 22/9; F = 18.79, p < 0.001) [87]. However, another study failed to report significant differences in arginase expression in platelets in subjects with bipolar disorder and controls [88]. Whilst the expression/activity of ornithine aminotransferase and ornithine transcarbamylase have not been specifically investigated in experimental and human studies of depressive or bipolar disorder, a recent study has reported that individuals with bipolar disorder had significantly higher plasma concentrations of spermine, a member of the polyamine family that is the end product of a series of enzymatic reactions that include ornithine decarboxylase, compared to healthy controls (879 ± 1021 vs. 174 ± 83 mmol/mL, p = 0.015) [89]. The hypothesis that alterations in ornithine concentrations in bipolar disorder reflect changes in the expression/activity of arginase and/or ornithine decarboxylase requires confirmation in further studies that should also investigate the activity of specific antidepressants on these enzymes. In this context, a study investigating the effects of antidepressant agents failed to demonstrate significant effects on arginase [90], whereas another reported a five-fold increase in ornithine decarboxylase activity with the tricyclic antidepressant imipramine [91]. Finally, the associations between ornithine and study continent are in line with previously reported differences in the concentrations of this metabolite associated with ethnicity [79].

A previously published systematic review and meta-analysis has investigated arginine, ornithine, and citrulline in major depressive disorder and reported no significant alterations of the three metabolites nor associations with pharmacological treatment [92]. Our meta-analysis captured a larger number of studies (20 vs. 13), which also included patients with bipolar disorders and the assessment of ADMA and SDMA, allowing a comprehensive investigation of the interplay between arginine metabolites with direct or indirect effects on NO synthesis and subtypes of mood disorder (depressive vs. bipolar).

Strengths of our work are the comprehensive assessment of arginine metabolomics in mood disorders (overall and by subtype), the investigation of potential correlations between the SMD and various parameters, and the robust assessment of the certainty of evidence and risk of bias. Limitations include the small number of articles, particularly studies investigating ADMA and SDMA. A further limitation is the high between-study heterogeneity observed. However, in subgroup analysis we identified potential sources of heterogeneity for ADMA (type of mood disorder), SDMA (study continent), citrulline (study continent), and ornithine (type of mood disorder, study continent, analytical method, and detection method).

In conclusion, our study has identified significant abnormalities in arginine metabolites and biomarkers of NO synthesis, i.e., ADMA, SDMA, citrulline, and ornithine, in subgroups of patients with mood disorders. These findings indicate a dysregulation of NO, particularly in bipolar disorder, and are hypothesis-generating for further research to unravel the pathophysiological role of arginine metabolism and NO and identify novel druggable targets in these patients.

## Data availability statement

The data that support the findings of this systematic review and meta-analysis are available from Angelo Zinellu upon reasonable request.

## Ethics declaration statement

Review and/or approval by an ethics committee was not needed as this is a systematic review and meta-analysis of published studies.

## CRediT authorship contribution statement

**Angelo Zinellu:** Writing – review & editing, Methodology, Formal analysis, Data curation, Conceptualization. **Sara Tommasi:** Writing – review & editing, Methodology, Conceptualization. **Stefania Sedda:** Writing – review & editing, Methodology. **Arduino A. Mangoni:** Writing – review & editing, Writing – original draft, Project administration, Methodology, Investigation, Conceptualization.

## Declaration of competing interest

The authors declare that they have no known competing financial interests or personal relationships that could have appeared to influence the work reported in this paper.

## Figures and Tables

**figs1:**
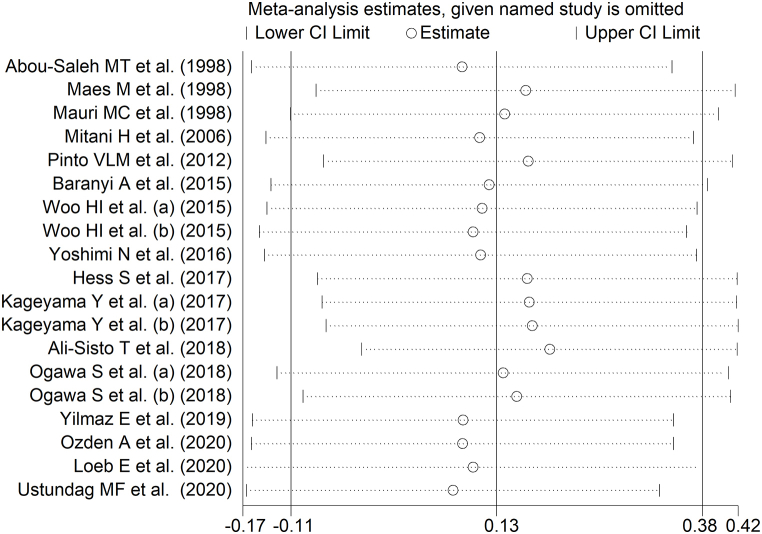


**figs2:**
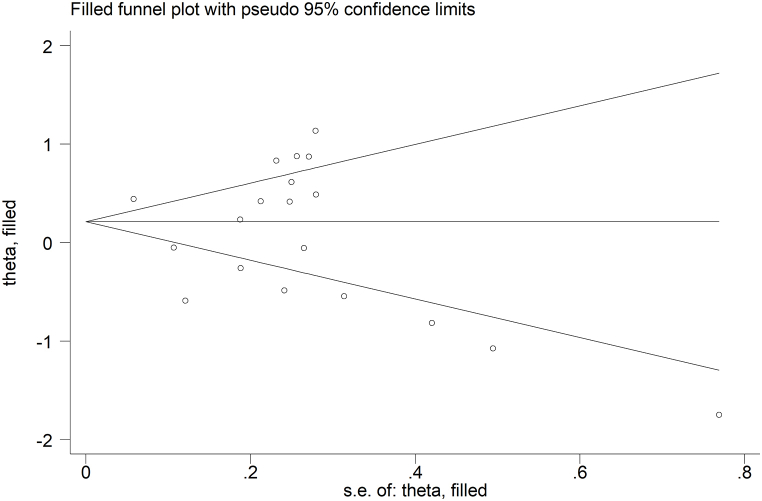


**figs3:**
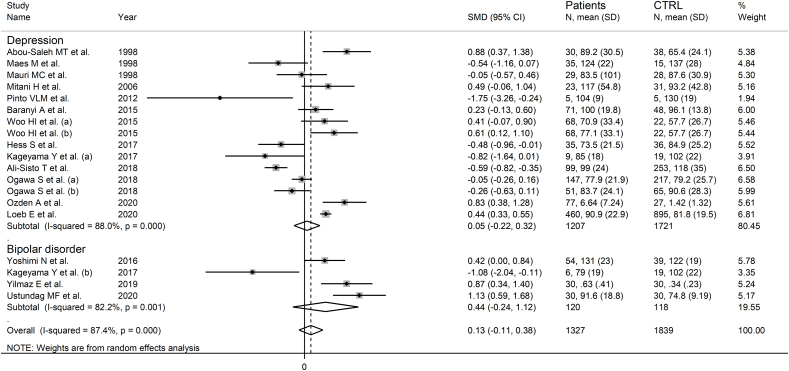


**figs4:**
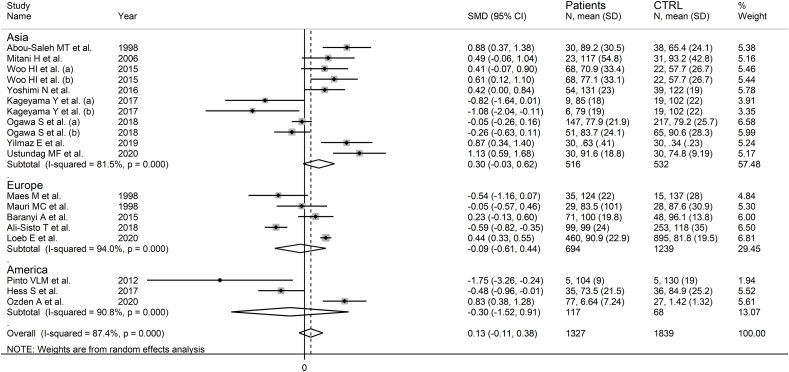


**figs5:**
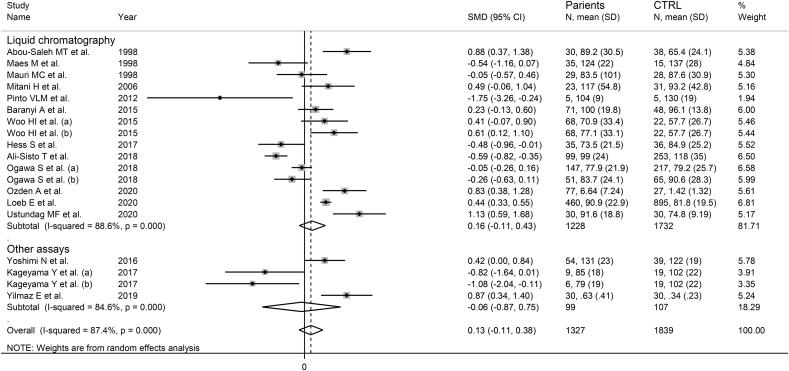


**figs6:**
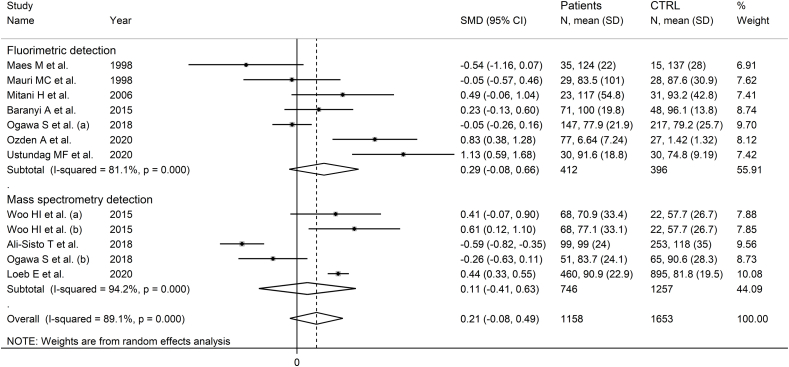


**figs7:**
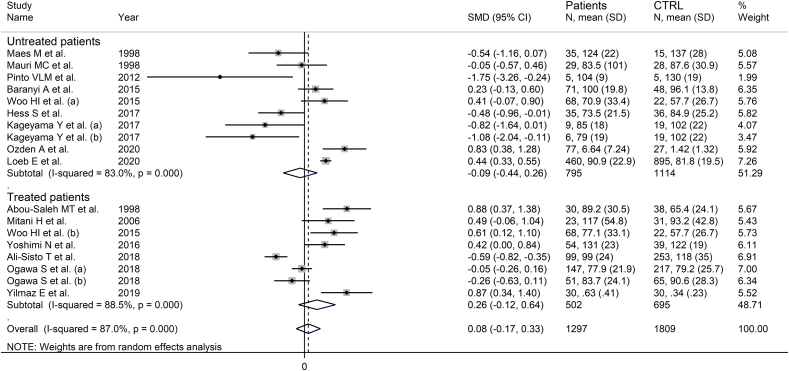


**figs8:**
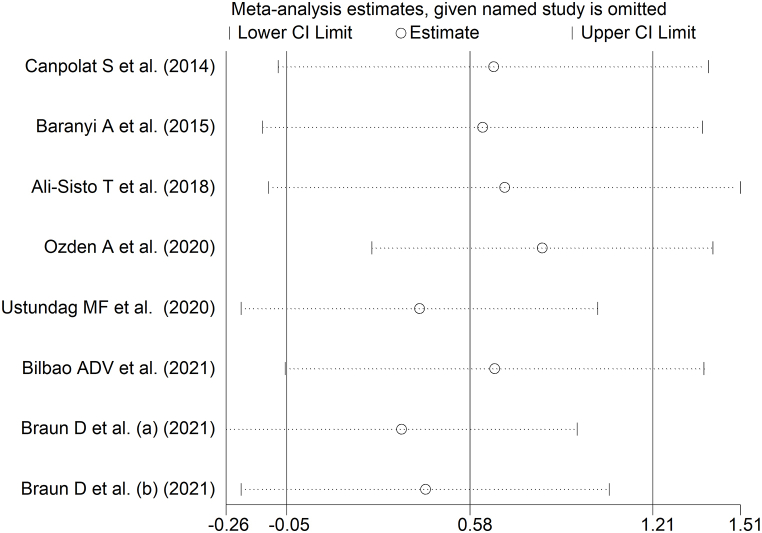


**figs9:**
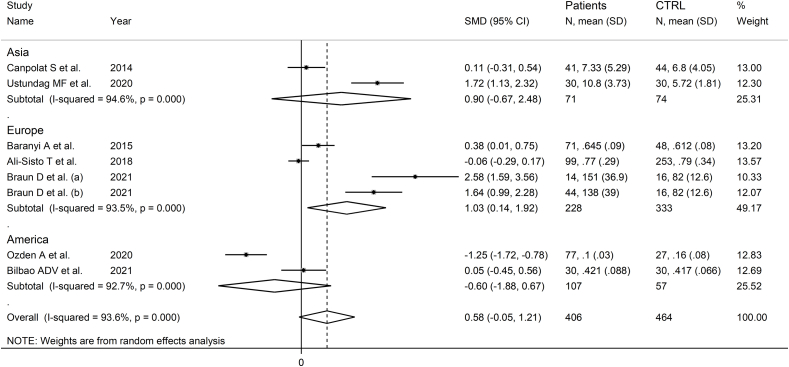


**figs10:**
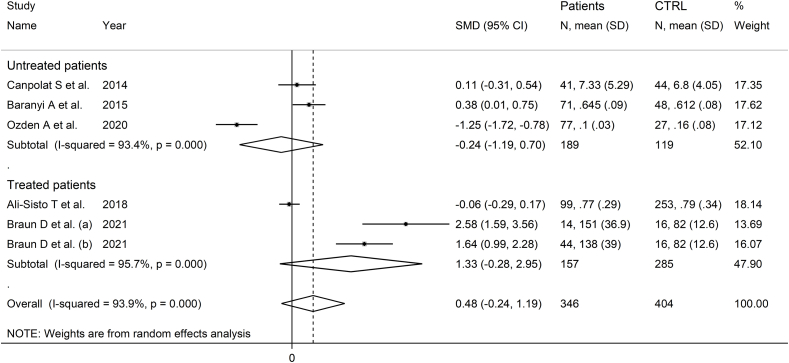


**figs11:**
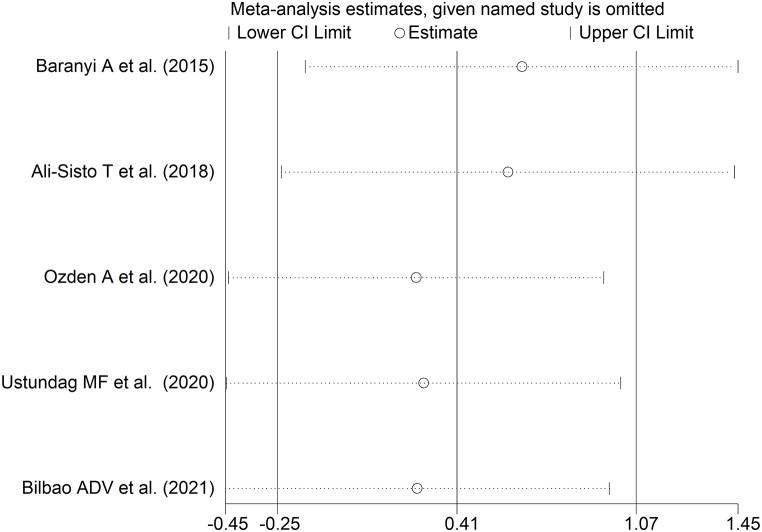


**figs12:**
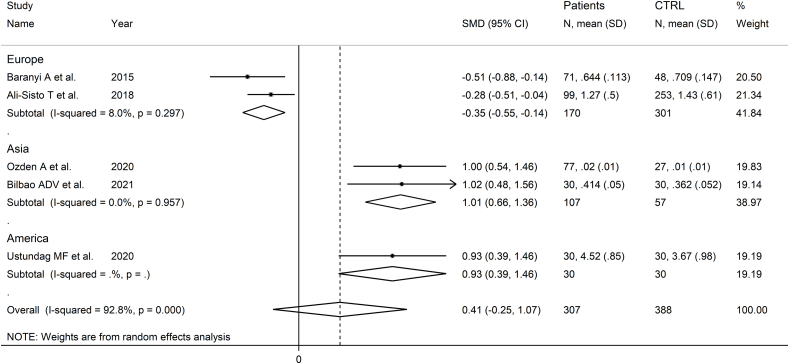


**figs13:**
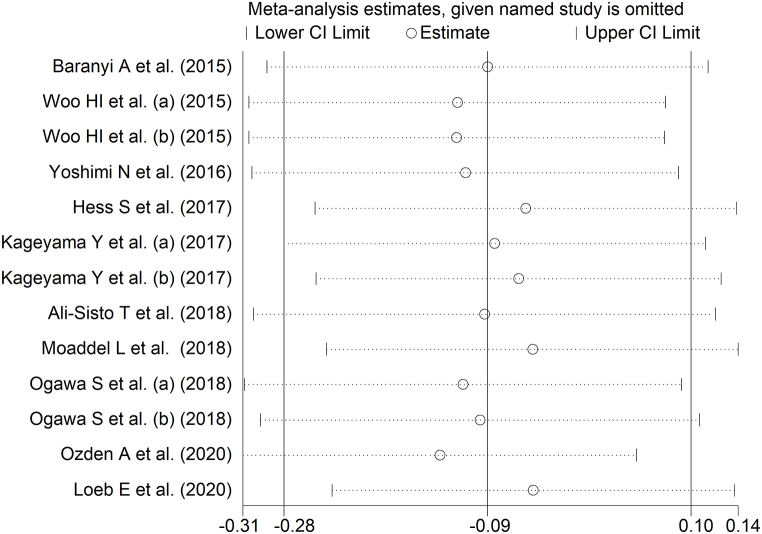


**figs14:**
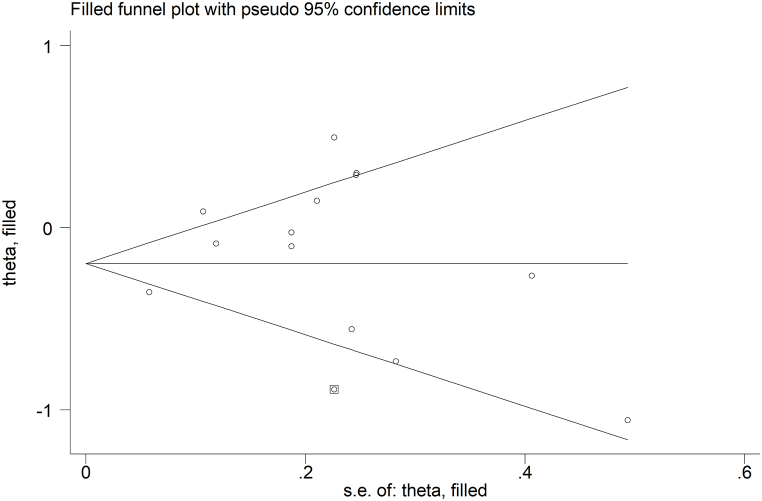


**figs15:**
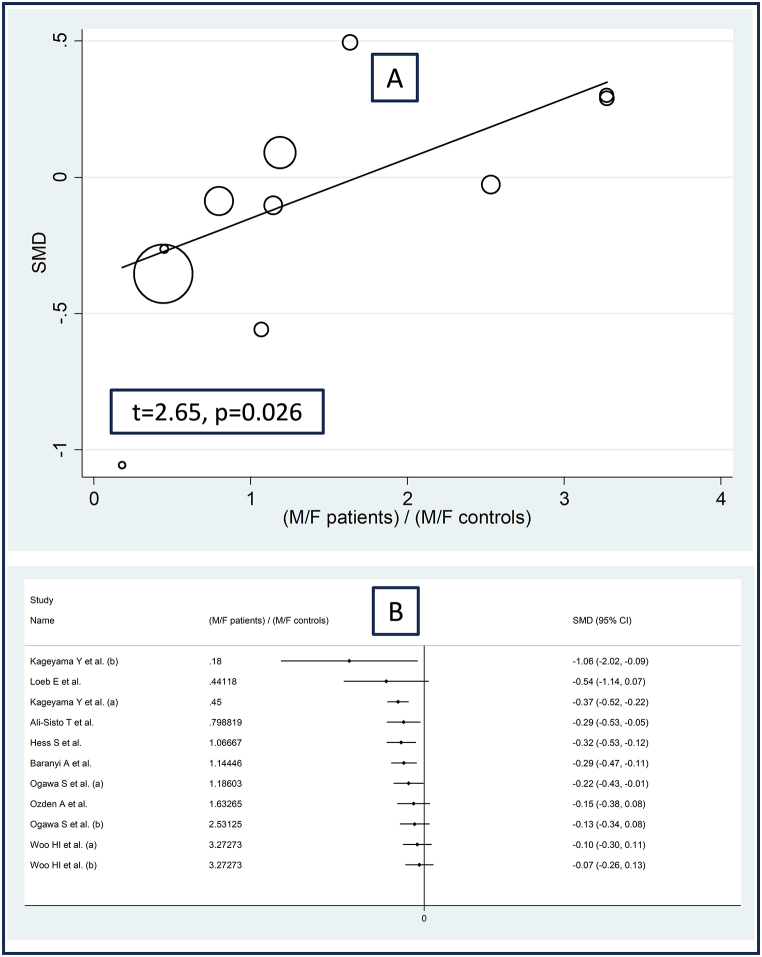


**figs16:**
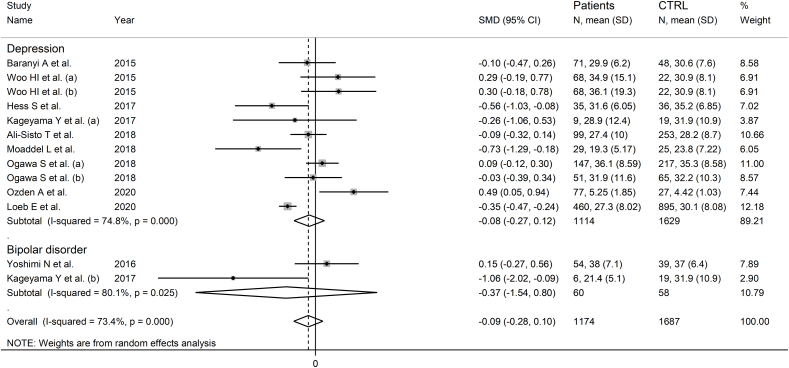


**figs17:**
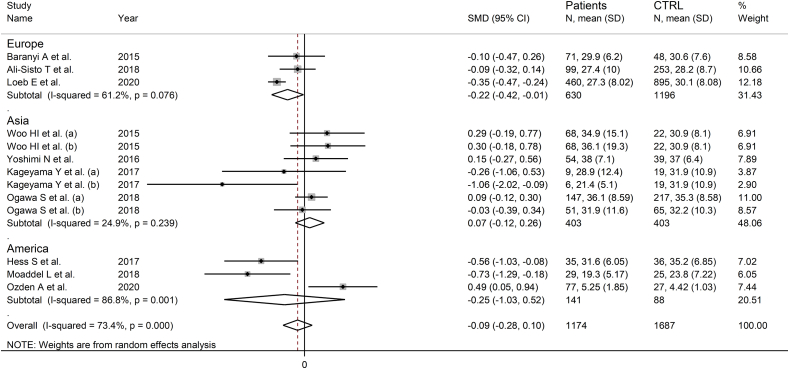


**figs18:**
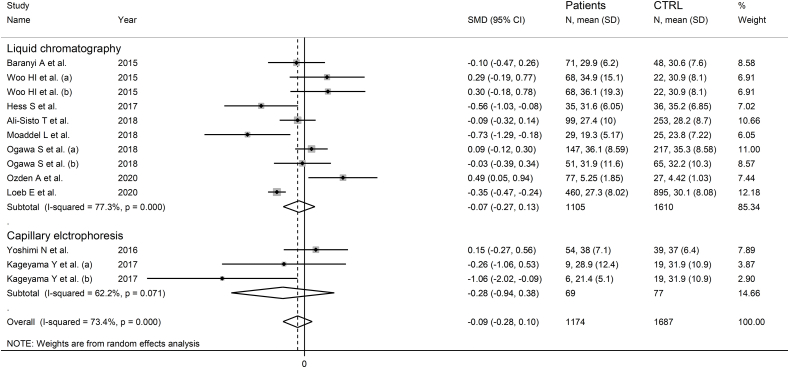


**figs19:**
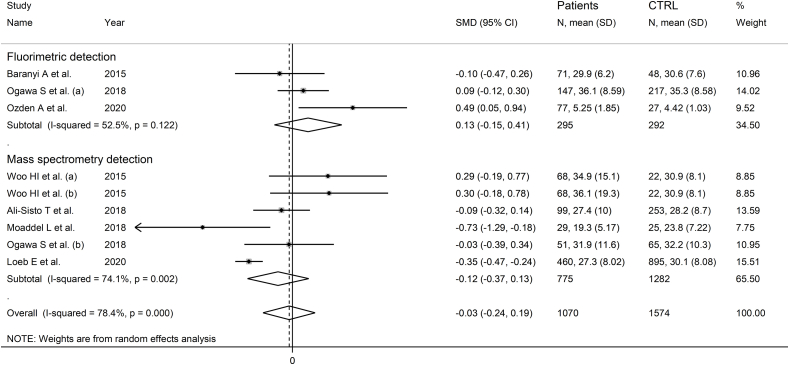


**figs20:**
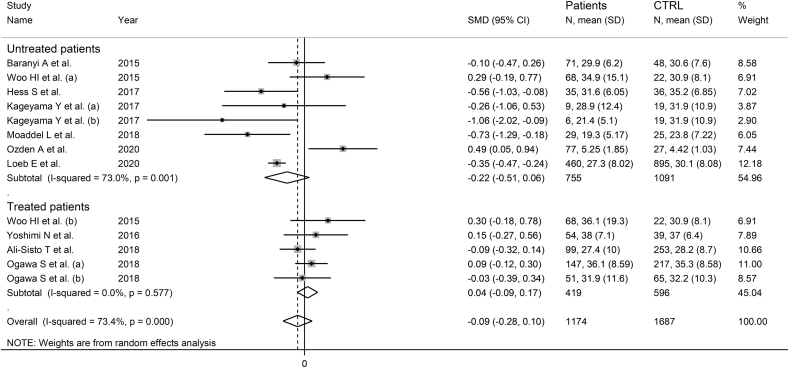


**figs21:**
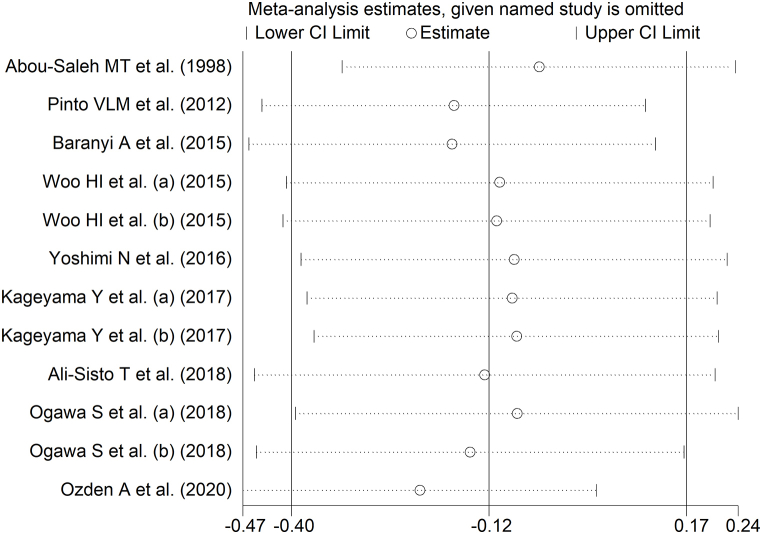


**figs22:**
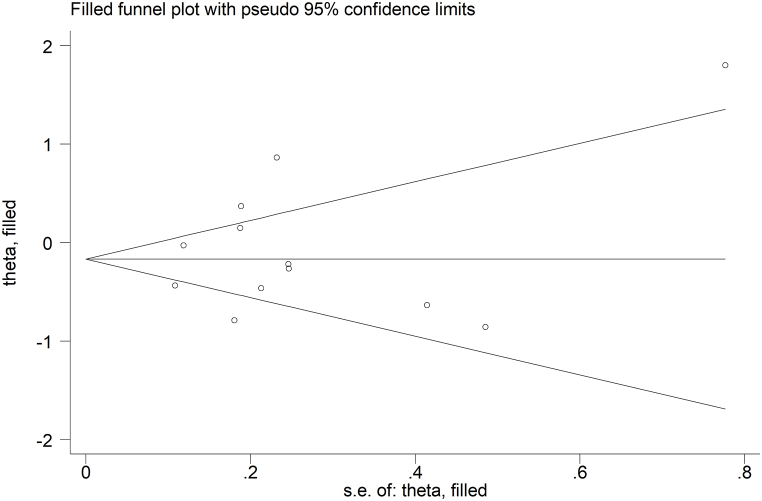


**figs23:**
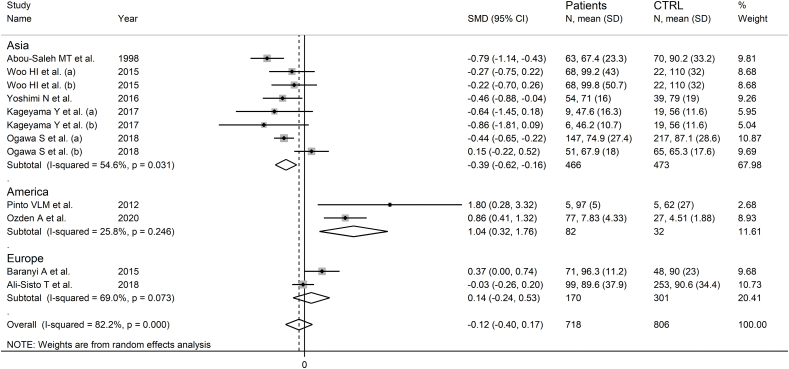


**figs24:**
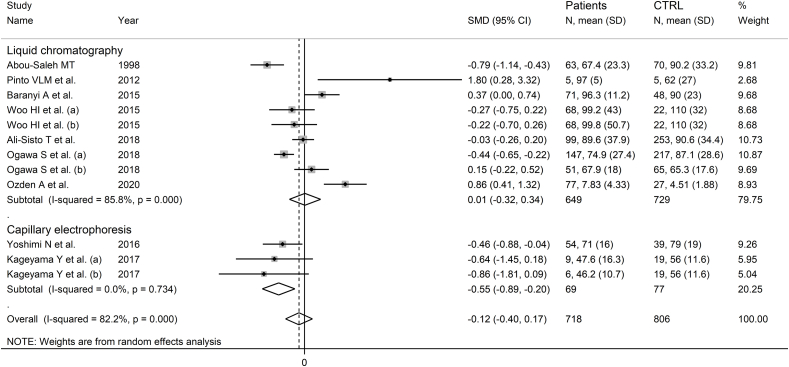


**figs25:**
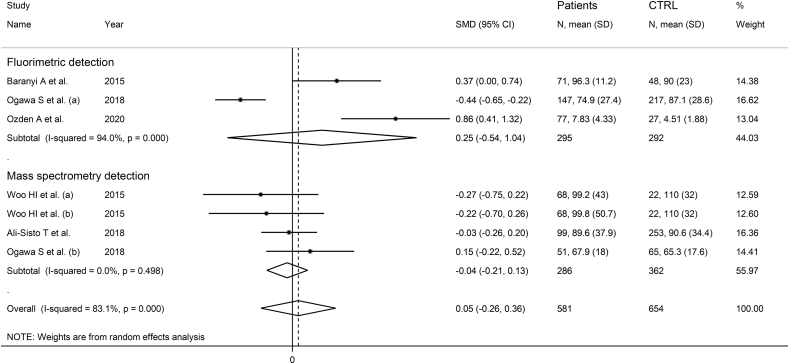


**figs26:**