# Supplementary material for: Arginine metabolomics in mood disorders
Source: Heliyon. 2024 Mar 14;10(6):e27292. doi: 10.1016/j.heliyon.2024.e27292 (PMC10955251; doi:10.1016/j.heliyon.2024.e27292)
Supplement: Multimedia component 2 [file mmc2.docx]

**Supplementary Table 2.** The Joanna Briggs Institute critical appraisal checklist.

| **Study** | **Were the criteria for inclusion clearly defined?** | **Were the subjects and the setting described in detail?** | **Was the exposure measured in a valid and reliable way?** | **Were objective, standard criteria used for measurement of the condition?** | **Were confounding factors identified?** | **Were strategies to deal with confounding factors stated?** | **Were the outcomes measured in a valid and reliable way?** | **Was appropriate statistical analysis used?** | **Risk of bias** |
| --- | --- | --- | --- | --- | --- | --- | --- | --- | --- |
| (Abou-Saleh et al., 1998) | No | No | Yes | Yes | Yes | Yes | Yes | Yes | Low |
| (Maes et al., 1998) | Yes | Yes | Yes | Yes | Yes | Yes | Yes | Yes | Low |
| (Mauri et al., 1998) | Yes | Yes | Yes | Yes | No | No | Yes | Yes | Low |
| (Mitani et al., 2006) | Yes | No | Yes | Yes | No | No | Yes | Yes | Moderate |
| (Pinto et al., 2012) | Yes | Yes | Yes | Yes | No | No | Yes | Yes | Low |
| (Canpolat et al., 2014) | Yes | Yes | Yes | Yes | No | No | Yes | Yes | Low |
| (Baranyi et al., 2015) | Yes | Yes | Yes | Yes | No | No | Yes | Yes | Low |
| (Woo et al., 2015) | Yes | Yes | Yes | Yes | Yes | Yes | Yes | Yes | Low |
| (Yoshimi et al., 2016) | Yes | Yes | Yes | Yes | Yes | Yes | Yes | Yes | Low |
| (Hess et al., 2017) | Yes | Yes | Yes | Yes | No | No | Yes | Yes | Low |
| (Kageyama et al., 2017) | Yes | Yes | Yes | Yes | Yes | Yes | Yes | Yes | Low |
| (Ali-Sisto et al., 2018) | Yes | Yes | Yes | Yes | Yes | Yes | Yes | Yes | Low |
| (Moaddel et al., 2018) | Yes | Yes | Yes | Yes | No | No | Yes | Yes | Low |
| (Ogawa et al., 2018) | Yes | Yes | Yes | Yes | Yes | Yes | Yes | Yes | Low |
| (Yilmaz et al., 2019) | Yes | Yes | Yes | Yes | No | No | Yes | Yes | Low |
| (Ozden et al., 2020) | Yes | Yes | Yes | Yes | Yes | Yes | Yes | Yes | Low |
| (Ustundag et al., 2020) | Yes | Yes | Yes | Yes | No | No | Yes | Yes | Low |
| (Bilbao et al., 2021) | Yes | Yes | Yes | Yes | No | No | Yes | Yes | Low |
| (Braun et al., 2021) | Yes | Yes | Yes | Yes | No | No | Yes | Yes | Low |
| (Loeb et al., 2022) | Yes | Yes | Yes | Yes | Yes | Yes | Yes | Yes | Low |
